# *Angiostrongylus cantonensis* Meningo‐Encephalitis in Children—Heightened Awareness Needed During Prolonged Wet Weather Conditions

**DOI:** 10.1111/jpc.16780

**Published:** 2025-01-17

**Authors:** Nadia Hasan, Clare Nourse, Claire Heney, Rogan Lee, Vishal Kapoor, Angela Berkhout

**Affiliations:** ^1^ Faculty of Medicine University of Queensland Brisbane Queensland Australia; ^2^ General Paediatrics The Queensland Children's Hospital Brisbane Queensland Australia; ^3^ Infection Prevention & Management Service The Queensland Children's Hospital Brisbane Queensland Australia; ^4^ Pathology Queensland Brisbane Queensland Australia; ^5^ NSW Health Pathology Centre for Infectious Diseases and Microbiological Services Westmead New South Wales Australia; ^6^ Westmead Clinical School, Faculty of Medicine and Health The University of Sydney, Westmead Hospital Westmead New South Wales Australia; ^7^ Departments of Paediatrics and Infectious Diseases Gold Coast University Hospital Southport Queensland Australia

**Keywords:** angiostrongylus, children, eosinophilic meningitis, neuroangiostrongyliasis

## Abstract

**Aim:**

*Angiostrongylus cantonensis,* the leading cause of eosinophilic meningoencephalitis, is well established in eastern Australia. Prolonged wet weather in Queensland during 2021–2022 coincided with anecdotal reports of increased neuroangiostrongyliasis cases, prompting an evaluation of paediatric cases from 2013 to 2022.

**Methods:**

This retrospective observational study reviewed children (0–16 years) with cerebrospinal fluid (CSF) eosinophilia (≥ 10% of the total CSF leukocyte count) and/or *A. cantonensis* testing (serology or polymerase chain reaction) between 01/01/2013 and 31/12/2022, using statewide laboratory data and patient records.

**Results:**

Eighty children were identified: 59 (74%) had CSF eosinophilia without *A. cantonensis* testing, 9 (11%) had CSF eosinophilia with *A. cantonensis* testing, and 12 (15%) had *A. cantonensis* testing without CSF eosinophilia. Neuroangiostrongyliasis was either proven or probable in seven children: five (71%) during 2021–2022, coinciding with prolonged wet weather. A significant positive correlation was observed between rainfall and case numbers (*r* = 0.88, *p* < 0.01). Median age of diagnosed children was 4 years (IQR 1.8–8.5, range 1.5–13 years) and five (71%) were male. Snail or slug exposure was reported in four (57%) children. All children presented with vomiting, and six also had a headache and focal neurology (86%). Abnormal neuroimaging was noted in six (86%) cases. Five children received corticosteroid therapy alone (71%), while two (29%) were managed conservatively. There were no deaths, but one child had persistent focal neurological abnormalities at discharge.

**Conclusion:**

Awareness of *A. cantonensis* and exposure risks is crucial, especially during prolonged wet weather conditions. While most children in this study had good outcomes, this is not always the case.

**Abbreviations**

*A. cantonensis*

:
*Angiostrongylus cantonensis*

ADEM: acute disseminated encephalomyelitis
B‐ALL: B‐cell acute lymphoblastic leukaemia
BRUE: brief resolved unexplained episode
CNS: central nervous system
CSF: cerebrospinal fluid
EVD: extra ventricular drain
ICP: intracranial pressure
IQR: interquartile range
LOC: level of consciousness
MOG: myelin oligodendrocyte glycoprotein antibody
MR: magnetic resonance imaging
PCR: polymerase chain reaction
SMA: spinal muscular atrophy
TBI: traumatic brain injury
VP shunt: ventriculoperitoneal shunt
WCC: white cell count

**Summary**

- What is known about this topic
  ○
*Angiostrongylus cantonensis* is well established in eastern Australia as the most common cause of eosinophilic meningoencephalitis.
  ○ Neuroangiostrongyliasis can result in severe morbidity or death, and its diagnosis requires a high index of clinical suspicion.
  ○ Prevention of this potentially devastating infection requires a high level of public awareness of the risks associated with mollusc ingestion and avoidance of exposure.
- What this paper adds
  ○ The majority of children with neuroangiostrongyliasis in Queensland between 2013 and 2022 presented during the years 2021–2022, with all cases coinciding with periods of prolonged wet weather.
  ○ Despite *A. cantonensis* being the leading cause of CSF eosinophilia, almost 90% of children with CSF eosinophilia were not tested for *A. cantonensis* , emphasising the need for improved clinician awareness and education on testing, especially during prolonged wet weather conditions.

## 1 Introduction

The rat lungworm, *Angiostrongylus cantonensis*, which is well established in Queensland and New South Wales, and has widely been reported as the most common cause of human eosinophilic meningo‐encephalitis [1, 2, 3, 4, 5, 6]. Neuroangiostrongyliasis can result in severe morbidity or death, and its diagnosis requires a high index of clinical suspicion. Prevention requires a high level of public awareness.

The parasitic nematode (roundworm) *A. cantonensis* likely originated in Southeast Asia but is now spreading widely throughout warmer, more humid parts of the world [7]. Adult worms live in the right ventricle and pulmonary arteries of several rat species, where they lay eggs that hatch and release first stage larvae (L1). L1s penetrate the alveoli, migrate to the pharynx, and are then swallowed, travelling through the digestive tract before being excreted in the rats' faeces. Once L1s enter the molluscan intermediate host through consumption of infected rat faeces, they undergo two moults to become infective third‐stage larvae (L3). Rats become infected by ingesting molluscs carrying these L3 larvae. In the rat's stomach, the larvae (L3) are released from the molluscs' tissues and migrate to the brain via the circulation. These L3 larvae (0.46–0.52 mm long, 0.22–0.27 mm wide) then undergo two further moults to become young adults (L5), before migrating to the pulmonary arteries where they mature and reproduce [3, 8]. The L5 larvae range from 9.7 to 11.5 mm, while mature adults reach 13–26 mm [9, 10].

Humans are accidental hosts, acquiring the infection by eating raw molluscs or paratenic hosts, either directly or masked in contaminated produce [11]. Once ingested, *A. cantonensis* are haematogenously transported to the central nervous system (CNS), burrowing into neural tissue. Usually, the young worms are unable to complete their life cycle in humans and die, leading to an intense granulomatous inflammatory response with a predominance of eosinophils [3]. However, instances of the worms migrating to the lungs have been reported, resulting in thrombi, pulmonary infarctions, and intra‐alveolar haemorrhages [12].

Infection severity depends on the parasitic burden and their movement within the CNS [1, 13, 14]. In humans, *Angiostrongylus* meningitis can be mild and self‐limiting with spontaneous resolution usually occurring within 6 weeks. However, cases can be severe, particularly in younger children and may result in meningoencephalitis, visual impairment, profound physical and intellectual disability, epilepsy, and death. Worse outcomes in younger children are likely due to ingestion of intact or multiple slugs or snails that are heavily infected [12, 13, 15].

Early diagnosis is challenging, as eosinophilia in blood and CSF are key features but may take weeks to develop and can fluctuate. While progress is being made to improve DNA detection, limitations in laboratory confirmation persist [16]. Symptomatic disease typically occurs between weeks one and two following infection, but parasite DNA detection and seroconversion may take several weeks [17, 18]. Clinicians need to have a high index of suspicion for neuroangiostrongyliasis in every case of eosinophilic meningitis, and a history of snail/slug ingestion should be actively sought.

Corticosteroids are the mainstay of treatment for neuroangiostrongyliasis, helping relieve symptoms and reduce CNS inflammation [19, 20]. Anthelminthics should only be used within the first three weeks post infection. They are not recommended beyond this period as when invading larvae in the CNS are killed, intense inflammation ensues and can exacerbate neurological symptoms. Antihelminthic treatment is considered likely to be most effective early, when larvae are small and yet to reach the CNS [21], and concomitant steroid treatment should be administered. Although the efficacy of prophylactic anthelminthic after mollusc ingestion has not been proven, Queensland and New South Wales guidelines currently recommend their use within 1–2 weeks following ingestion [22, 23].

During 2021 and 2022, increased rainfall and flooding in Queensland [24] coincided with anecdotal reports of rising neuroangiostrongyliasis cases. Floods typically displace rats to higher ground, while rain brings molluscs to surface areas and vegetation, potentially heightening exposure risk. The Infection Management and Prevention Service at the Queensland Children's Hospital provides state‐wide Infectious Diseases advice and noted a potential rise in cases during this period. This study subsequently sought to review the Queensland incidence, disease characteristics and outcomes of neuroangiostrongyliaisis cases in children using state‐wide clinical and laboratory data over a 10‐year period.

## 2 Methods

Ethics approval for this study was obtained from the Children's Health Queensland Hospital and Health Service (Queensland Children's Hospital) Human Research Ethics Committee (HREC/23/QCHQ/95673).

### 2.1 Inclusion Criteria

Children aged 0–16 years with (CSF) eosinophilia (≥ 10% of the total CSF leukocyte count) and/or *A. cantonensis* testing performed (serology or polymerase chain reaction (PCR)) between 1/1/2013 and 31/12/2022 were identified from statewide laboratory data. Cases of either probable or proven neuroangiostrongyliasis were further evaluated.

### 2.2 Case Definitions

Laboratory confirmation of *A. cantonensis* infection was defined as detection of *A. cantonensis* by PCR in CSF or seroconversion in blood or CSF, along with eosinophilic meningitis.

Proven neuroangiostrongyliasis was defined as laboratory confirmation of *A. cantonensis* with one or more of the following: lethargy, fever (≥ 38°C), headache, seizures, focal neurology or abnormal neuroimaging.

Probable neuroangiostrongyliasis was defined as symptomatic disease with eosinophilic meningitis, without laboratory confirmation, and no alternate diagnosis.

### 2.3 Case Ascertainment and Data Extraction

Children (0–16 years) with *A. cantonensis* testing performed and/or CSF eosinophilia (as defined above) were identified from statewide laboratory records, between 1st January 2013 up to and including 31st December 2022.

Patient records, where available, were reviewed for details of age, sex, potential risk factor/source of infection (known snail/slug ingestion, ingestion of unwashed produce), clinical features, medical history, laboratory findings, alternative diagnoses, treatment (steroids, anthelminthic use) and outcome at discharge (focal neurology, death).

### 2.4 Statistical Analyses

Descriptive analyses were conducted for clinical characteristics and outcomes. Categorical variables were presented as proportions, while continuous variables were described using means and standard deviations (SD) for normally distributed data or medians with interquartile ranges for non‐normally distributed data. Spearman's correlation and Poisson regression analyses were conducted to evaluate associations between the number of annual neuroangiostrongyliasis cases and both time (in years) and total annual rainfall in Queensland, using data from the Australian Bureau of Meteorology. Data were analysed using Stata 18 statistical software (StataCorp. 2023. College Station, TX).

## 3 Results

**FIGURE 1:**
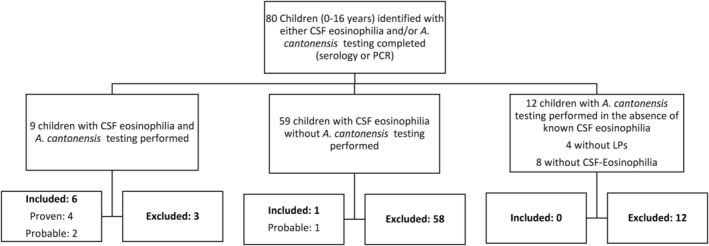
Overview of the children identified with either CSF eosinophilia and/or children who had *A. cantonensis* testing performed. CNS, Central nervous system; CSF, Cerebrospinal fluid; EVD, Extra ventricular drain; ICP, Intracranial pressure; PCR, Polymerase chain reaction.

Eighty children (0–16 years) were identified with either CSF eosinophilia and/or *A. cantonensis* testing (Figure 1). Of the 59 children with CSF eosinophilia but no *A. cantonensis* testing, one was classified as a probable neuroangiostrongyliasis case. The classification was made despite treating the clinical team's diagnosis of presumptive viral meningitis, based on the clinical presentation, CSF eosinophilia (20%), and the absence of an alternate diagnosis known to be associated with CSF eosinophilia. The remaining 58 children were given an alternative diagnosis by their treating clinical team (Table 1). Amongst the seven neuroangiostrongyliasis cases (four confirmed, three probable), the median age was four years (IQR 1.8–8.5, range 1.5–13 years), with a male (71%) predominance (Table 2).

**TABLE 1:** Excluded Queensland children with CSF eosinophilia and/or *A. cantonensis* testing performed (2013–2022).

**Children with CSF eosinophilia with *A. cantonensis* testing performed (*n* = 3)**	
Autoimmune encephalitis	
ADEM—MOG positive	2
CNS infection	
Cysticercosis	1
**Children with CSF eosinophilia without *A. cantonensis* testing performed (*n* = 58)**	
Intraventricular device insitu – routine testing performed	17
Intraventricular device dysfunction/revision	(12)
Inserted in the setting of CNS malignancy	3
Inserted for other reasons	9
Intraventricular device insitu; routine testing without known dysfunction. Inserted in the setting of:	(5)
CNS malignancy	1
Acquired brain injury	1
To facilitate cerliponase administration for Batten's disease	1
Congenital aqueduct stenosis	1
Traumatic brain injury	1
CNS Infection	12
Bacterial meningitis	(4)
*Escherichia coli*	1
Methicillin‐susceptible *Staphylococcus aureus*	1
Group B streptococcus	1
Culture negative	1
Viral meningitis	(2)
Enterovirus	1
Cytomegalovirus	1
Ventriculitis	(6)
*Enterobacter cloacae*	1
*Staphylococcus epidermidis*	1
*Escherichia coli*	1
Culture negative	3
Non‐CNS infection	12
Bacterial	(2)
Group B streptococcus bacteraemia	1
*Escherichia coli* pyelonephritis	1
Viral	(2)
Rhinovirus	1
Bronchiolitis with an afebrile seizure – unidentified virus	1
Parasitic	(2)
Congenital toxoplasmosis	2
Fever without an identified organism	(6)
Neonate (0–4 weeks)	2
Infant (4–12 weeks)	3
Infant (3–12 months)	1
Malignancy and or related complication	5
Acute myeloid leukaemia with CNS involvement – diagnostic testing	1
B‐ALL—intrathecal chemotherapy administration	1
Graft versus host disease with peripheral eosinophilia	1
Pilocytic astrocytoma (following surgical resection)	1
Pilocytic astrocytoma (noted at EVD insertion)	1
Other	12
Neonatal BRUE/apnoea/lethargy	3
Autoimmune encephalitis – ADEM – MOG positive	2
SMA – CSF sampled with nusinsersin administration	2
Chiari malformation – noted at ventriculoperitoneal shunt insertion	1
Hypoxic ischaemic encephalopathy	1
Incontinentia pigmenti, post‐ictal	1
Neonatal pustular lesions	1
Transferred to a private hospital – further information not available	1
**Children who were tested for *A. cantonensis* in the absence of CSF eosinophilia (*n* = 8)**	
CNS infection	(2)
Pachymeningitis	1
CNS fungal infection – *Debaryomyces hansenii*	1
CNS inflammation	(3)
Autoimmune encephalitis – ADEM – MOG positive	1
Acute haemorrhagic leukoencephalopathy	1
CNS vasculitis	1
Other	(3)
Transferred to a private hospital – further information not available	1
Lab error – case initially mistakenly identified as CSF eosinophilia	1
Peripheral eosinophilia in the absence of other symptoms	1
**Children who were tested for *A. cantonensis* in the absence of a lumbar puncture (*n* = 4)**	
Prolonged fever (3 weeks) without an alternative diagnosis identified	1
Anterior uveitis in the context of travel to Indonesia	1
Peripheral eosinophilia without an alternative diagnosis identified	1
Foreign traveller with influenza A	1

Abbreviations: *A. cantonensis* , *Angiostrongylus cantonensis* ; ADEM, Acute disseminated encephalomyelitis; B‐ALL, B‐cell acute lymphoblastic leukaemia; BRUE, brief resolved unexplained episode; CNS, central nervous system; CSF, cerebrospinal fluid; MOG, myelin oligodendrocyte glycoprotein antibody; SMA, spinal muscular atrophy.

**TABLE 2:** Characteristics of the proven and probable neuroangiostrongyliasis cases in children (0–16 years) between 2013 and 2022 in Queensland, Australia.

Year	Age, years	Gender	Geographical source of infection	Exposures	Clinical presentation	CSF WCC × 10^6^/L; eosinophil %	Neuroimaging (MRI Brain +/− Spine)	CSF serology	Serum serology	CSF PCR	Treatment	Outcome
2016^a^	4	Male	Western Downs Region	Unknown	Fever, headaches, vomiting, focal neurology, seizures, Altered LOC	45; 20	Normal	Not done	Not done	Not done	Conservative management	No adverse outcome
2017^a^	5	Female	Recent visit to Bali	Snails in garden	Headache, vomiting	307; 58	Tubular structures over posterior left cerebellar hemisphere	Negative	Negative	Not done	Conservative management	No adverse outcome
2021^a^	12	Male	Brisbane	Unknown	Headache, vomiting, focal neurology, lethargy	72; 70	Left superior parietal lobule and precuneus hyperintense, partially enhancing lesions. Leptomeningeal disease.	Negative	Negative	Not done	Corticosteroids	Focal neurology—Lower limb hypoesthesia
2022^b^	2	Male	Brisbane	Snail stuck in nose	Fever, headache, vomiting, focal neurology, lethargy	149; 20	Acute right peri‐trigonal infarction	Negative	Equivocal	Positive	Corticosteroids	No adverse outcome
2022^b^	13	Male	Gold Coast	Home grown lettuce	Headache, vomiting, focal neurology	980; 57	Right corona radiata and basal ganglia focal linear hyperintensity.	Equivocal	Equivocal	Positive	Corticosteroids	No adverse outcome
2022^b^	1.5	Male	Brisbane	Snails at home	Vomiting, focal neurology, lethargy	680; 25	Bilateral foci of cortical diffusion restriction with mild increase in pial enhancement and bilateral papilloedema.	Equivocal	Positive	Positive	Corticosteroids	No adverse outcome
2022^b^	1.5	Female	Sunshine Coast	Unknown	Fever, headache, vomiting, focal neurology, lethargy	310; 53	Leptomeningitis affecting brain and spine. Some small punctate cortical parenchymal changes.	Equivocal	Positive	Positive	Corticosteroids	No adverse outcome

Abbreviations: CSF, cerebrospinal fluid; LOC, level of consciousness; MR, magnetic resonance imaging; PCR, polymerase chain reaction; WCC, white cell count.

^a^ Probable cases.

^b^ Proven cases.

### 3.1 Geographic Distribution and Impact of Rainfall on Neuroangiostrongyliasis Cases (Table 2)

A statistically significant positive correlation was observed between annual rainfall and neuroangiostrongyliasis cases (Spearman's rho = 0.75, *p* = 0.02). However, multivariable Poisson regression analysis using rainfall and year as predictor variables revealed no significant associations. All cases occurred during multiyear La Niña events in 2016–2017 and 2020–2022 and notably, 71% of cases occurred during 2021 and 2022 [25]. All of the six cases acquired in Queensland, Australia, were acquired in South East Queensland and surrounding areas, with three (50%) from Brisbane, two (33%) from surrounding coastal cities and one (17%) from the nearby Western Downs region. The seventh case was likely acquired in Indonesia.

### 3.2 Risk Factor and Exposure History (Table 2)

Exposures to snails were reported in three children (43%). One child had a history of a snail being lodged in their nostril approximately 3 months prior to presentation. Although this incident is unlikely to be directly responsible for this presentation, as it does not align with known incubation periods, it suggests a potential predisposition to further snail exposure. Another child had consumed homegrown lettuce, which was presumed to be contaminated by snails or slugs.

### 3.3 Clinical Presentation and Investigations (Table 2)

At presentation, vomiting was reported in all children; focal neurology, and headaches were reported in 86% of children. Lethargy or altered level of consciousness was reported in five (71%) and fever in three (43%). Four children (57%) required multiple presentations to emergency departments before being admitted to hospital for further evaluation and subsequent diagnosis.

### 3.4 Management and Outcomes

Five children (71%) were managed with corticosteroids (three received a 7‐day course, two a 14‐day course, both followed by a tapering regimen), while the remaining two (29%) children, both of whom were considered probable cases, were conservatively managed. Anthelminthics were not used in any of the cases.

The median duration of hospital admission was eight days, with no admissions to the intensive care unit. Six (86%) children had no adverse outcomes, while one child (14%) had hypoesthesia in the lower leg at discharge. Four children (57%) re‐presented to the hospital within three weeks of discharge with on‐going symptoms, and one of them received a second corticosteroid course. The two children who received a 14‐day corticosteroid course did not re‐present with on‐going symptoms.

## 4 Discussion

All described neuroangiostrongyliasis cases in Queensland occurred during periods of prolonged wet weather, a pattern also observed in Hawaii and Mayotte [26, 27], where increased rainfall correlated with higher infection rates in gastropods [28, 29]. Similar peaks in incidence have also been noted in canine cases in Eastern Australia during periods of increased humidity [30] and wet weather (Rivory P et al. Rainfall and temperature‐driven emergence of neural angiostrongyliasis in eastern Australia 2020–2024, manuscript under review). Seasonal variations also depend on local intermediate host populations, for example, in Tahiti, freshwater prawn abundance influenced case numbers, while in New Caledonia, native molluscs played a similar role [30]. Although studies on the seasonality of *A. cantonensis* infection in humans are limited, evidence suggests that weather events influence the prevalence of this parasite in rat populations. Wet weather displaces rats to higher ground, encourages molluscs up to the surface and to breed more, thereby heightening the exposure risk [31]. However, the exact duration or amount of rainfall required to increase infection risk remains unknown. This association highlights the importance of maintaining a higher index of clinical suspicion, particularly during and after significant wet weather.

The youngest child in this study with neuroangiostrongyliasis was 18‐months old, yet cases in infants as young as nine months have been reported [32]. It is probable that younger infants face a lower risk of infection due to their limited mobility, but once children become mobile, especially during their oral sensory‐seeking phase, they may become particularly vulnerable to accidental ingestion.

Notably, 59 children (87%) with CSF eosinophilia did not undergo *A. cantonensis* testing, including one with probable neuroangiostrongyliasis (Tables 1 and 2). Nearly 30% of these children had intraventricular devices at the time of CSF testing, with samples collected for routine monitoring rather than suspected infection. In such cases, CSF eosinophilia is commonly seen, potentially due to shunt dysfunction or hypersensitivity to foreign materials [33, 34, 35]. Twenty‐four (41%) children were managed for alternative CNS or non‐CNS infections, including two children diagnosed with congenital toxoplasmosis, a recognised cause of CSF eosinophilia [36]. Two children with culture negative ventriculitis, following neurosurgical procedures, may have experienced CSF eosinophilia due to hypersensitivity to foreign materials. The remaining children were managed for confirmed or presumptive bacterial or viral infections. While bacterial and viral CNS infections can occasionally cause CSF eosinophilic pleocytosis, this is not common [37]. Mild CSF eosinophilia can be associated with primary CNS tumours, leukaemia and lymphoma with CNS involvement, and graft versus host disease [37, 38, 39], as observed in five children in this study. Four children were diagnosed with myelin oligodendrocyte glycoprotein (MOG) antibody‐associated acute disseminated encephalomyelitis, a condition known to cause CSF eosinophilia in about one‐third of patients [40]. CSF eosinophilia was noted in two children receiving antisense oligonucleotide therapy for spinal muscular atrophy and one with a Chiari malformation. These cases were not further investigated due to the absence of other concerning features, although an association with CSF eosinophilia in these conditions has not been reported. Six neonates presented with CSF eosinophilia without *A. cantonensis* testing. One was diagnosed with incontinentia pigmenti, a condition associated with eosinophilia [41]. In the remaining neonates, the discharge diagnosis did not explain the CSF eosinophilia, although *A. cantonensis* infection is unlikely at this age. Over half (53%) of the children with CSF eosinophilia without *A. cantonensis* testing had blood‐stained CSF, with > 1000 red blood cells × 10^6^/L. While no data suggests that traumatic CSF samples lead to an overestimation of eosinophils, this possibility warrants further investigation.

While the above proposed alternative diagnoses may explain some of the children with CSF eosinophilia without *A. cantonensis* testing, it is possible that some were in fact attributable to *A. cantonensis* . It is recognised that most children with neuroangiostrongyliasis experience a mild illness that resolves without treatment, however, given the potential for severe outcomes, including death and neurological sequelae, increased awareness amongst clinicians and the public is essential [4]. Clinician education on appropriate testing (serology and PCR for both CSF and serum) and post‐exposure albendazole prophylaxis following mollusc ingestion is critical [22, 23]. Consultation with local infectious diseases teams in cases of CSF eosinophilia can facilitate prompt *A. cantonensis* testing. It is important to note that *A. cantonensis* testing is only available at a few specialist laboratories worldwide. In Australia, testing is referred to The Institute of Clinical Pathology and Medical Research Centre for Infectious Diseases and Microbiology at Westmead Hospital. Additionally, *A. cantonensis* antibodies and DNA may not be detectable in the early stages of infection, and negative results do not rule out neuroangiostrongyliasis. In such cases, repeat serum serology should be considered, and consultation with infectious diseases teams is recommended.

While only one child with neuroangiostrongyliasis experienced on‐going neurological morbidity and most had a favourable outcome, this is not always the case. A 1‐week course of corticosteroids is generally sufficient when indicated [42], although longer courses may be necessary for children with persistent symptoms, as observed in one child in this study. Although anthelminthics were not used for any of the children, they may be used in some cases, and the decision should be made on a case‐by‐case basis, considering factors such as time of exposure. However, it is recommended that corticosteroids be given concurrently with anthelminthics when used to treat neuroangiostrongyliasis [17].

A strength of this study was the comprehensive case ascertainment using state‐wide laboratory data. Limitations included its retrospective observational design and inclusion of probable cases, without diagnostic confirmation. This study may also underestimate the burden of neuroangiostrongyliasis in Queensland children, as milder cases may have been missed due to an alternative diagnoses or lack of CSF or *A. cantonensis* testing. In this study, CSF eosinophilia was defined as eosinophils comprising at least 10% of the total CSF leukocyte count, a threshold based on a single study [43]. However, the normal eosinophil count in CSF is not well established due to limited research on normal CSF parameters in healthy individuals [44]. Additionally, while rainfall data from all of Queensland were used, cases were concentrated in the South East Queensland and surrounding regions, where rainfall patterns may differ from those across the state. However, the numbers were too small to do sub‐analysis to assess associations between the rainfall patterns in different geographical regions and cases of *A. cantonensis* in these regions. Finally, long‐term neurological outcomes were not assessed and should be addressed in future studies.

## 5 Conclusion

Children are more likely to present with *A. cantonensis* meningo‐encephalitis during prolonged wet weather conditions. Clinician and public awareness of this parasite, risk of mollusc exposure and significance of CSF eosinophilia is important.

## Ethics Statement

Ethics approval for this study was obtained from the Children's Health Queensland Hospital and Health Service (Queensland Children's Hospital) Human Research Ethics Committee (HREC/23/QCHQ/95673).

## Conflicts of Interest

The authors declare no conflicts of interest.
